# Supplementary material for: PP2Ac Modulates AMPK-Mediated Induction of Autophagy in Mycobacterium bovis-Infected Macrophages
Source: Int J Mol Sci. 2019 Nov 29;20(23):6030. doi: 10.3390/ijms20236030 (PMC6928646; doi:10.3390/ijms20236030)
Supplement: Supplementary file 1 [file ijms-20-06030-s001.pdf]

International Journal of
Molecular Sciences

Supplementary Materials:

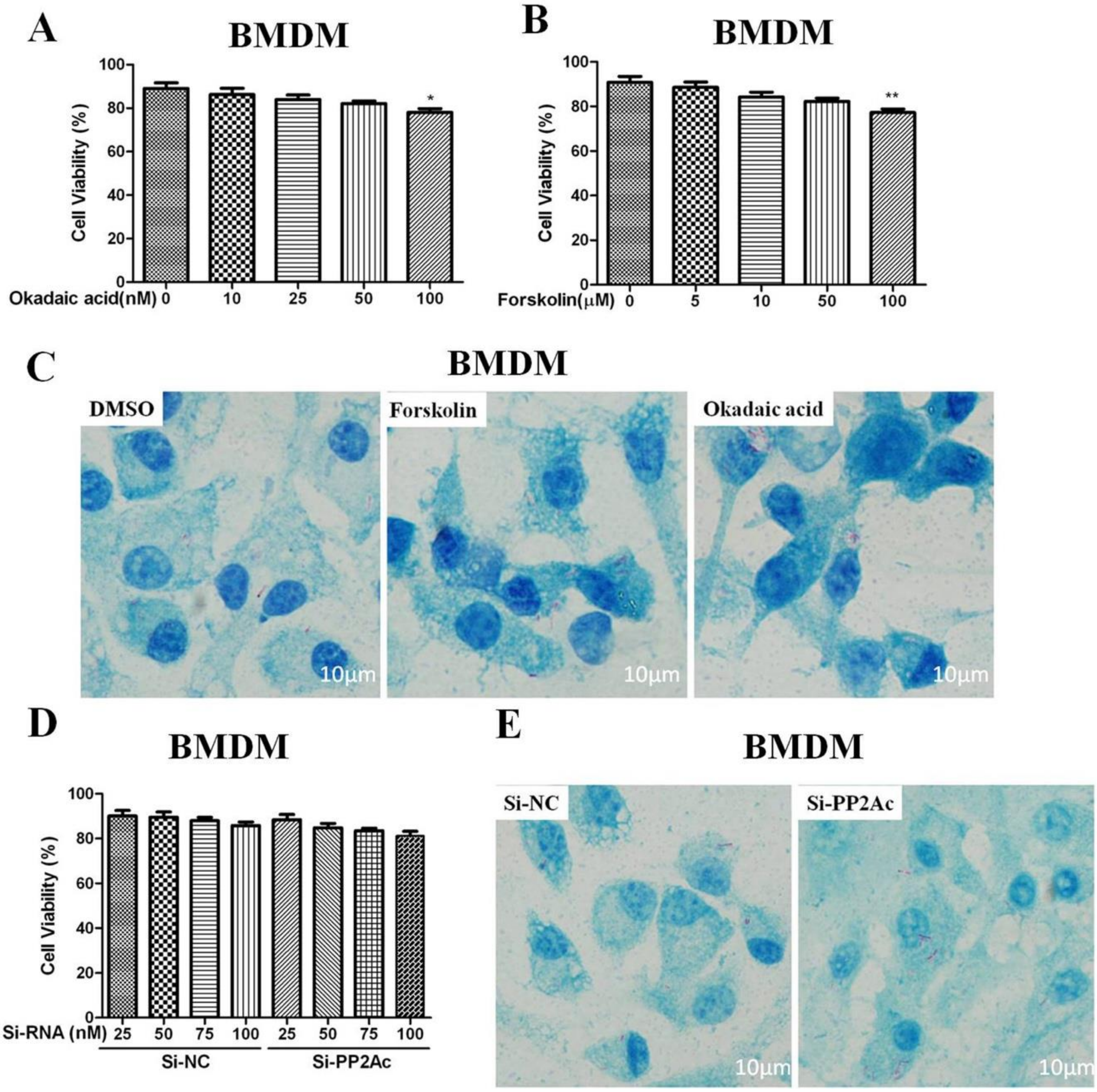

Supplementary Figure1. Cell viability assay and phagocytic ability of macrophages infected with *M. bovis*. (A) BMDM cells were cultured in 96-well plates for 12 to 18 hours and then treated with different concentration of (A) okadaic acid and (B) forskolin. Cells viability was assessed by measuring Optical density (OD) after addition of MTS reagent with an ELISA plate reader. (C) BMDM cells were cultured on small cover glasses in 24-well plates then treated with DMSO (0.1%), okadaic acid and forskolin followed by *M. bovis* infection (MOI 1:10). After infection cells were stained for acid fast bacilli and observed under a microscope. (D) BMDM cells were cultured in 96-well plates for 12 to 18 hours and then treated with different concentration of Si-NC and Si-PP2Ac for 36 hours. Cells viability was assessed by measuring Optical density (OD) after addition of MTS reagent with an ELISA plate reader. (E) BMDM cells were cultured on small cover glasses in 24-well plates then treated with different concentration of Si-NC and Si-PP2Ac for 36 hours followed by *M. bovis* infection. Cells were stained for acid fast bacilli and observed under microscope. Scale bar: 10μm. Data represent the mean ± SD from three independent experiments. (*$p<0.05$, **$p<0.01$)

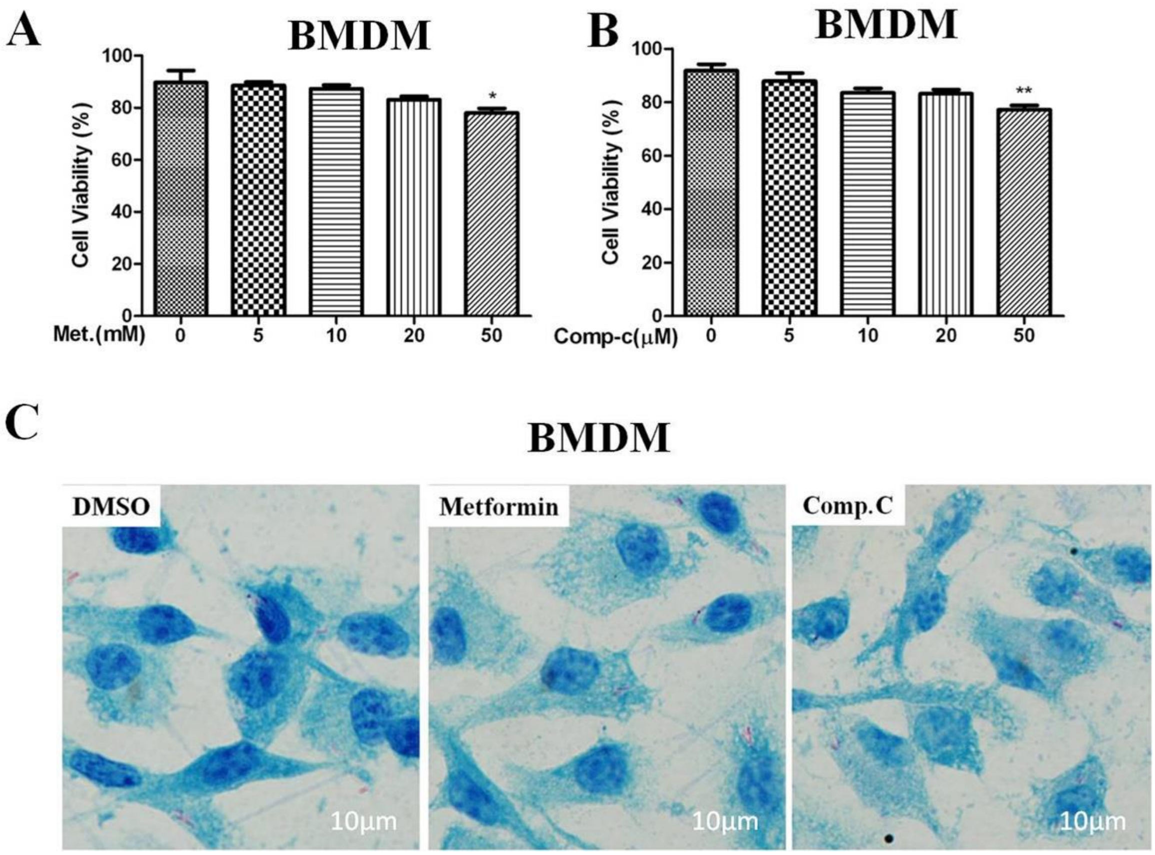

Supplementary Figure 2. The effect of metformin and compound-c on cell viability or phagocytic ability of infected macrophages. (A) BMDM cells were cultured in 96-well plates for 12 to 18 hours and then treated with different concentration of (A) metformin and (B) compound-c. Cells viability was assessed by measuring Optical density (OD) after addition of MTS reagent with an ELISA plate reader. (C) BMDM cells were cultured on small cover glasses in 24-well plates then treated with DMSO (0.1%), metformin and compound-c followed by *M. bovis* infection (MOI 1:10). After infection cells were stained for acid fast bacilli and observed under a microscope. Scale bar: 10 μm. Data represent the mean ± SD from three independent experiments. (**p*<0.05, ***p*<0.01)
